# Polymorphisms near the *IFNL3* Gene Associated with HCV RNA Spontaneous Clearance and Hepatocellular Carcinoma Risk

**DOI:** 10.1038/srep17030

**Published:** 2015-11-25

**Authors:** Mei-Hsuan Lee, Hwai-I Yang, Sheng-Nan Lu, Yu-Ju Lin, Chin-Lan Jen, Kang-Hsuan Wong, Soa-Yu Chan, Liang-Chun Chen, Li-Yu Wang, Gilbert L’Italien, Yong Yuan, Chien-Jen Chen

**Affiliations:** 1 Institute of Clinical Medicine, National Yang-Ming University, Taipei, Taiwan; 2 Genomics Research Center, Academia Sinica, Taipei, Taiwan; 3 Division of Hepato-Gastroenterology, (Department of Internal Medicine), Kaohsiung Chang Gung Memorial Hospital, KaohsiungChang Gung University School of Medicine, Kaohsiung, Taiwan; 4 Graduate Institute of Microbiology and Immunology, National Yang-Ming University, Taipei, Taiwan; 5 Faculty of Medicine, National Yang-Ming University, Taipei, Taiwan; 6 MacKay Medical College, Taipei, Taiwan; 7 Global Health Economics and Outcomes Research, Bristol-Myers Squibb, Princeton, NJ, United States; 8 Yale University School of Medicine, New Haven, CT, United States; 9 Graduate Institute of Epidemiology and Preventive Medicine, College of Public Health, National Taiwan University, Taipei , Taiwan

## Abstract

The aims of this study were to investigate associations between single nucleotide polymorphisms (SNPs) near the genes *IFNL2*, *IFNL3*, and *IFNL4* and spontaneous clearance of hepatitis C virus (HCV) and to evaluate variants for their risk of hepatocellular carcinoma (HCC) among subjects in whom spontaneous HCV RNA clearance did not occur. In the first study, 889 untreated anti-HCV-seropositive patients without HCC symptoms were followed from 1991 to 2005. The spontaneous HCV clearance rate was found to be 33.1%. The TT variant of rs8099917 near *IFNL3* was associated with increased spontaneous HCV RNA clearance, with an adjusted odds ratio (95% CI) of 2.78 (1.43–5.39), as was the newly-identified TT/TT dinucleotide variant rs368234815 near *IFNL4* (adjusted odds ratio 2.68, 95% CI: 1.42–5.05). In the second study, associations between SNPs and HCC risk were examined in 483 HCC cases with detectable HCV RNA and 516 controls. In participants with HCV genotype 1, unfavorable genotypes for HCV clearance near *IFNL3*were associated with increased HCC risk, the adjusted odds ratio (95% CI) for rs12979860 and rs8099917 being 1.73 (1.00–2.99) and 1.84 (1.02–3.33), respectively. Host characteristics should be considered to identify high-risk patients to prioritize the use of new antiviral agents and intensive screening.

Hepatitis C virus (HCV) infects more than 170 million people worldwide (approximately 3% of the human population)^1^. It has been estimated that HCV is responsible for one third of cases of hepatocellular carcinoma (HCC) globally, representing a great public health burden^2^. Chronic HCV- infected subjects with detectable HCV RNA have elevated risks for HCC and liver-related deaths^3^,^4^. In addition to liver diseases, individuals with detectable HCV may have high risks of extra-hepatic diseases^4^,^5^.

Spontaneous clearance of HCV RNA occurs in approximately 8–36% of chronic hepatitis C patients without antiviral treatment^6^,^7^,^8^,^9^,^10^,^11^,^12^,^13^. Previous studies showed that subjects with a black ancestry are less likely to have spontaneous resolution of HCV than other ethnic groups^6^,^7^,^8^, suggesting that a host genetic basis might be associated with viral clearance. Recent genome-wide association studies have identified single nucleotide polymorphisms (SNPs) near the *IFNL3* (formerly *IL28B*) gene, located on the long arm of chromosome 19, that are associated with treatment-induced RNA clearance in chronic hepatitis C patients who receive conventional interferon-based therapy^14^,^15^,^16^. In addition to treatment-induced RNA clearance, the same genetic polymorphisms were shown to have impacts on spontaneous clearance of HCV RNA^17^,^18^,^19^. There are great differences in *IFNL3* polymorphisms between various ethnic populations and, in contrast to Caucasians, most Asians carry favorable *IFNL3* genotypes and have better interferon treatment responses^19^. Whether the *IFNL3* polymorphisms are associated with HCV spontaneous clearance in Asians at the community level needs further investigations.

The results of several recent studies examining the association of *IFNL3* variants and liver diseases have been inconsistent^20^,^21^,^22^,^23^,^24^,^25^,^26^,^27^. In one study, no association between *IFNL3* variants and the risk for HCC was found after adjustment for clinical parameters^28^, although this could be due to the small number of HCC cases examined, resulting in insufficient statistical power. We previously showed that patients with detectable HCV RNA have an increased risk for HCC^3^ and should be considered as requiring antiviral therapy. Among these high-risk patients, it is important to know whether host variants are associated with HCC, as this would help identify patients who need to be treated with the new expensive antiviral agents (interferon-free based). Screening for the variants rs8099917 and rs12979860 is already frequently used in the clinic to predict the patient’s response to antiviral treatment, and, if the host variants are also associated with liver disease progression, this information would help stratify the high risk patients and prioritize them for intensive care and the utility of this testing would be enhanced.

The aims of this study were therefore to examine associations between genetic polymorphisms near the interferon-lambda genes, *IFNL2*, *IFNL3*, and *IFNL4* and HCV RNA spontaneous clearance among patients in the community and to evaluate the effect of these polymorphisms on the risk for HCC. The two most frequently examined candidate SNPs near*IFNL3*, rs12979860 and rs8099917 were examined, since individuals who carry the TT genotype of rs8099917 or the CC genotype of rs12979860 have better treatment responses and these genotypes are considered as favorable^14^,^15^,^16^. Both of the two SNPs were located on the intergenic region and it is needed to narrowing the signal to potentially causative variants. Thus we genotyped as more as known SNPs in the region as possible to determine whether one of these SNPs have a stronger association with HCV RNA clearance or HCC. In addition, the newly discovered dinucleotide variant, rs368234815, near^29^ *IFNL4* was included.

## Materials and Methods

### Study participants, data collection, and laboratory examinations

#### (i)For the study of associations between genetic polymorphisms near the interferon-lambda genes IFNL2, IFNL3, and IFNL4 and HCV RNA spontaneous clearance

In this study, 23, 820 random individuals aged 30–65 years who had previously been enrolled from seven townships in Taiwan during 1991–1992 for the Risk Evaluation of Viral Load Elevation and Associated Liver Disease/Cancer (R.E.V.E.A.L) study^3^,^30^ were examined and 1095 were found to be seropositive for anti-HCV antibodies, but seronegative for HBsAg; none had received antiviral treatment and none showed signs of HCC. Of these, 889 had adequate samples for serum HCV RNA tests and SNP genotyping and were included in the analyses of HCV spontaneous clearance. All the participants gave their written informed consent for the interview, health examination, and blood collection. Participants seropositive for antibodies against HCV (anti-HCV) or hepatitis B surface antigen (HBsAg) at study entry were regularly followed up by abdominal ultrasonography and health examinations until the end of 2005.This study was approved by the Institutional Review Board of the College of Public Health, National Taiwan University, Taipei, Taiwan and was carried out in accordance with the approved guidelines. The details of the study participants, interview, blood collection and laboratory examinations have been described previously in detail^3^,^30^.

The participants were interviewed by study nurses using a structured questionnaire including information on demographic characteristics, personal history of major diseases, and lifestyle habits. In addition, each participant provided a 10 ml blood sample taken by a standardized procedure and serum samples were prepared on the same day and stored at −70 °C until assay. Serostatus for HBsAg and anti-HCV antibodies was determined, and serum levels of triglycerides, total cholesterol, alanine aminotransferase (ALT), and aspartate aminotransferase (AST) measured, using commercial kits as described previously^3^. Serum levels of HCV RNA were measured using the COBASTaqMan HCV test, version 2 (Roche Diagnostics, Indianapolis, NJ) with a detection limit of 25 IU/mL.

#### (ii) For the case-control study to examine the risk for HCC associated with SNPs significantly associated with spontaneous HCV RNA clearance

The 595 participants with detectable HCV RNA at the end of the above study were included in the study to evaluate associations between genetic variants and the risk for HCC. The 79 participants who developed HCC during follow-up in the above study served as cases, while the 516 who did not served as controls. In order to obtain sufficient statistical power, we also included 404 HCC samples from the Taiwan Liver Cancer Network (TLCN)^31^,^32^ with detectable HCV RNA to increase the number of cases. The TLCN is a biobank which contains DNA samples from HCC patients from most of the major medical centers in Taiwan and the samples can be taken as representative of all HCC cases in Taiwan. HCC patients were diagnosed by pathological or clinical evidence (image plus elevated α fetoprotein levels) in the medical center and invited to provide written informed consent for the collection and storage of their biospecimen samples. All of the DNA samples were stored at −80 °C until tested. We therefore compared the distributions of the candidate SNPs in 483 HCC cases and 516 controls.

### Detection of spontaneous HCV RNA clearance

The baseline serum samples of the study participants were retrieved for HCV RNA tests. In the case of participants with detectable serum HCV RNA levels at baseline, we also retrieved their samples from the last follow-up for additional HCV RNA tests. Because the infection time is unknown among individuals who acquired HCV in in the community, it is not practical to follow-up patients regularly to define their incidence of HCV RNA clearance. Thus the definition of HCV spontaneous clearance was less rigorous. Participants who had undetectable HCV RNA at baseline and participants who had detectable HCV RNA levels at baseline, but undetectable levels at the last follow-up were classified as showing spontaneous HCV RNA clearance. In total, 294 of the 889 study participants showed spontaneous clearance of HCV RNA and 595 did not.

### Genotyping of single nucleotide polymorphisms near the *IFNL2*, *IFNL3*, and *IFNL4* genes

Genomic DNA was extracted using QIAGEN commercial kits and a standardized protocol. The frequently examined SNPs, rs8099917 and rs12979860, which have been found to be associated with HCV treatment responses in previous genome-wide association studies^14^,^15^,^16^, are located between IFNL2 (formerlyIL28A) and IFNL3 (formerlyIL28B). These genes are of moderate size (1.3–1.5 kb) and are separated by a distance of 23 kb. In 2013, another gene, IFNL4, was discovered 29 and is located in the 23 kb region between IFNL2 and IFNL3. In this study, we genotyped 73 SNPs in a 32,500 base pair region that included IFNL2, IFNL3, and IFNL4s and their regulatory regions. We utilized Illumina VeraCode GoldenGate Genotyping assay, which is a flexible and customized platform for genotyping. We made a region file which provided information regarding to the region we would like to study. The file contained the SNPs which might be available in the specified region for the probe design. Genotyping of all SNPs except rs368234815 at IFNL4 was performed using the Illumina VeraCode GoldenGate Genotyping Assay, a flexible and customized platform, while the TT/TT, TT/ΔG, and ΔG/ΔG variants were later genotyped using the TaqMan assay29.

### Statistical analysis

The baseline characteristics of age, sex, body mass index, serum levels of triglycerides and total cholesterol, and serum levels of AST and ALT were examined to evaluate their associations with spontaneous HCV RNA clearance using the chi-squared test. The crude and multivariate odds ratios (ORs) and the 95% confidence intervals (CIs) for these associated factors were estimated to evaluate the magnitude of their correlation with spontaneous RNA clearance by logistic regression.

Genetic markers following the Hardy-Weinberg equilibrium and with a minor allele frequency of >1% were included in the analyses. The Hardy-Weinberg equilibrium of each marker was examined using the chi-squared test. Correlations between an SNP and the linkage disequilibrium block containing the SNP were displayed using the HAPLOVIEW program (http://www.broadinstitute.org/haploview)^33^. The association between the SNP genotype and HCV RNA spontaneous clearance was assessed using the chi-squared test. In addition, the false discovery rate procedure was used to correct for multiple comparisons^34^. SNPs found to be significant (with corrected p values < 0.05) in this test were included in the multiple logistic regression model to estimate the odds ratio (OR) and 95% CI for their association with HCV RNA clearance.

SNPs significantly associated with viral clearance were examined for a relationship with the risk for HCC in participants with detectable HCV RNA. The SNPs of the cases were compared to those in the controls. Adjusted ORs and the 95% CIs were generated after adjustment for potential confounders. In addition, the HCV genotype was stratified as genotype 1 and genotype non-1 to test the association of genotype with risk for HCC. All statistical procedures were performed using SAS software (version 9.3; SAS Institute Inc, Cary, NC).

## Results

### Spontaneous HCV RNA clearance rate

In this part of the study, of the 889 community-based participants seropositive for anti-HCV antibodies, but seronegative for HBsAg, who had not received antiviral treatment and showed no signs of HCC, 294 showed spontaneous HCV RNA clearance, i.e. the RNA clearance rate was 33.1%. For the group as a whole, females had a higher HCV RNA spontaneous clearance rate than males (40.0% vs. 24.5%, p < 0.001), there was no obvious trend between age and HCV RNA clearance rate, and the HCV clearance rate was 38.2%, 36.8%, 29.8%, and 31.0% for individuals aged 30–39, 40–49, 50–59, and ≥60 years old, respectively (data not shown).

### Determinants of HCV RNA spontaneous clearance

Table 1 shows the distribution of the baseline characteristics of these 889 participants stratified by spontaneous HCV RNA clearance or persistence. Females, obese subjects (BMI ≥ 23 kg/m^2^), and those with elevated serum triglyceride and total cholesterol levels were more likely to show spontaneous HCV RNA clearance (p < 0.05), as were participants with decreased serum ALT or AST levels (p < 0.001).

Table 2 shows the ORs (95% CI) for the baseline characteristics and their association with spontaneous HCV RNA clearance. After adjustment for other relevant determinants, females, obese participants, and those with high serum levels of triglyceride and total cholesterol were more likely to show spontaneous HCV RNA clearance. Compared to males, females had an adjusted OR of 1.99 (1.43–2.77) for spontaneous HCV RNA clearance, the adjusted OR (95% CI) for participants with a serum triglyceride level ≥150 mg/dl was 2.17 (1.50–3.15) compared to those with a triglyceride level of <150 mg/dl, and that for participants with a total cholesterol level of ≥240 mg/dl was 2.46 (1.47–4.13) compared to those with a total cholesterol level of <240 mg/dl. Participants with elevated ALT or AST serum levels were less likely to show spontaneous HCVRNA clearance (p < 0.001 using the Cochran-Armitage trend test).

### SNPs near the *IFNL2*, *IFNL3*, and *IFNL4* genes

**Figure 1:**
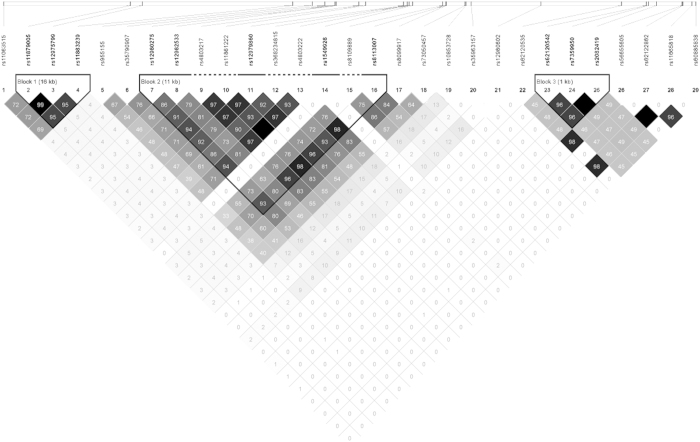
Positions and correlations (presented as r^2^) of single nucleotide polymorphisms near the *IFNL2*, *IFNL3*, and *IFNL4* genes.

Of the 73 SNPs genotyped, 25 showed no genetic variation in our population, 15 had a minor allele frequency less than 1%, and 4 violated Hardy-Weinberg equilibrium and were excluded from analyses. In our population, most individuals carried the favorable genotype of rs8099917 and rs12979860, with 91.7% having the TT genotype of rs8099917 and 90.1% the CC genotype of rs12979860. For the dinucleotide variation of rs368234815, 91.4% of individuals carried TT/TT. A higher proportion of individuals with spontaneous HCV RNA carried the homozygous genotypes of the SNPs examined. The positions and correlations of the 29 SNPs are shown in Fig. 1. Three blocks could be differentiated within the region. The correlation between rs8099917 and rs12979860 was 0.81 and these two SNPs were in the same block. The r^2^ for the association of rs368234815 with rs8099917 or rs12979860 was, respectively, 0.76 and 0.92.

### Association between these SNPs and spontaneous HCV clearance

These 29 SNPs were then tested for associations with spontaneous viral clearance (Supplementary Table 1). As shown in Table 3, after multiple corrections by the false discovery rate, 11 SNPs showed significant differences in distribution between participants who showed spontaneous HCV RNA clearance and those who did not (p < 0.05).

Table 4 shows the ORs and 95% CIs for the associations between these11 SNPs and spontaneous HCV RNA clearance. Individuals who carried the homozygous genotypes had a higher probability of showing spontaneous HCV RNA clearance. The crude ORs for the eleven SNPs ranged from 1.93 to 3.00 and allele even remained significantly associated with spontaneous HCV RNA clearance after considering potential confounders. The adjusted OR and 95% CI was 2.53 (1.43–4.49) for rs12979860, 2.29 (1.24–4.21) for rs8099917, and 2.68 (1.42–5.05) for rs368234815.

### Case-control study to detect associations between these 11 SNPs and HCC risk

The 11 SNPs significantly associated with HCV RNA spontaneous clearance were then examined for a relationship with HCC in a case-control study in which the cases consisted of the 79 subjects from the REVEAL study who developed HCC and 404 HCC cases from the TLCN study, while the controls were the 516 subjects from the REVEAL study who did not develop HCC. As shown in Table 5, after stratifying by HCV genotype, rs8099917 and rs12979860 were found to be associated with risk of HCC among the participants with HCV genotype 1 infection. After adjustment for age, sex, and serum levels of ALT and HCV RNA, there was still significant associations between rs8099917 and rs12979860and HCC, the adjusted OR being, respectively, 1.84 and 1.73. However, rs368234815 was not significantly associated with HCC among patients with HCV genotype 1 or genotype non-1. Participants who carried the minor allele of the SNPs examined, who were less likely to show spontaneous HCV RNA clearance, had an increased risk of HCC.

## Discussion

In this community-based study, the HCV spontaneous clearance rate was 33.1%, which is relatively high compared to that seen in other countries^6^,^7^,^8^,^9^,^10^,^11^,^12^,^13^. Some previous studies defined spontaneous HCV RNA clearance by following individuals with detectable HCV RNA and measuring HCV RNA levels periodically^7^,^8^,^12^. However, it is not practical to follow-up asymptomatic HCV infected patients in the community and, in Taiwan, iatrogenic risk factors account for 80% of HCV infection and the infection time is unknown^35^. In our study, we therefore defined spontaneous HCV RNA clearance as (i) undetectable RNA at baseline or (ii) detectable RNA at baseline, but undetectable RNA in the last test and our definition was therefore less rigorous than in some other studies. In addition, the great genetic variation near *IFNL3* (*IL28B*) in different ethnic populations might explain the variation in HCV clearance rate in different countries^19^.

Our study showed, consistent with previous studies^13^,^19^, that the *IFNL3* genotype was associated with spontaneous HCV RNA clearance. The frequency of the favorable allele for rs8099917 (T) was 0.96 and that for rs12979860 (C) was 0.95. These two SNP markers were highly linked (r^2^ = 0.81), suggesting that only one needs to be tested to predict a patient’s treatment response. Other genetic variants have been found to be associated with spontaneous clearance of HCV infection^36^,^37^,^38^. A recent genome-wide association study showed that, in addition to SNPs near the *IFNL3* gene, several SNPs near genes for HLA class II molecules may also affect spontaneous HCV clearance^17^. It will be interesting to validate the SNPs found in other studies in our population^17^,^36^,^37^,^38^ and examine the gene-gene interactions associated with HCV spontaneous clearance.

Consistent with other studies^13^,^39^,^40^, we found that females had a higher HCV RNA spontaneous clearance rate. Among untreated chronic hepatitis C patients, serum HCV RNA levels are lower in women than in men^41^. Sex hormones might contribute to the gender-difference in clearance rate. Estradiol was found to act directly on the virus by having an inhibitory effect on the release of mature virions from HCC cell lines^42^. Pregnancy, a state with elevated estrogen and progesterone, was found to decrease the activity of chronic HCV infection^43^. Our findings are consistent with those of a previous large-scale community-based study, which showed that the HCV RNA-positive rate is higher in patients with normal serum triglyceride or total cholesterol levels than in those with abnormal levels^44^. HCV and free low density lipoprotein compete for the lipoprotein receptor on cells and it has been suggested that free beta-lipoproteins in human serum might regulate the rate of HCV infection of liver cells^45^. We therefore hypothesize that high concentrations of triglyceride and total cholesterol during HCV infection may compete with HCV for binding to hepatocyte receptors, allowing the virus to be cleared more easily.

SNPs rs12979860 and rs8099917, which have been frequently examined in studies of associations with HCV RNA clearance^13^,^14^,^15^,^16^,^19^, are located between the *IFNL2* and *IFNL3* genes. In order to find causal variants, we examined SNPs in a 32 kb region containing the *IFNL2* and *IFNL3* genes (previously, *IL28A* and *IL28B*) and later shown to contain the *IFNL4* gene^29^,^46^. We then narrowed down the region to 12,410 base pairs which contained 11 SNPs that were significantly associated with HCV spontaneous clearance. Interestingly, some of these SNPs have been found to be associated with the response to interferon-based treatment in are-sequencing and fine-mapping study^47^. SNP rs4803217 is in the 3′ untranslated region (UTR) of the *IFNL3* locus, while rs4803222 is located in the 5′ UTR of a newly identified interferon gene *IFNL4*^29^,^46^. *In vitro* study has shown that the G allele of rs48303217is associated with decreased degradation of *IFNL3* mRNA^48^, and this polymorphism has been proposed as a causal variant improving HCV clearance. Our findings are consistent with those of recent studies showing that patients carrying the *IFNL4*-ΔG allele have less likelihood of showing spontaneous or treatment-induced HCV clearance^49^,^50^. Expression of IFN-λ4 protein differs inpatient with different *IFNL4*-ΔG genotypes^29^, suggesting that the polymorphism is highly functional. Recently, it was found that patients with serine, rather than proline, at position 70inIFN-λ4 display lower hepatic interferon-stimulated gene expression and better spontaneous HCV clearance^51^.

In addition to spontaneous RNA clearance, we assessed the associations between the 11polymorphismsnear *IFNL3* and *IFNL4* that were associated with spontaneous clearance and the risk of HCC among individuals with detectable HCV RNA (i.e. those not showing spontaneous HCV RNA clearance).In our analysis, we found that, among patients infected by HCV genotype 1, those who carried the alleles associated with less likelihood of spontaneous RNA clearance had an increased risk for HCC. This finding is consistent with the results of a previous study which showed that the T allele of rs12979860 (unfavorable allele) increases the risk for HCC recurrence among patients who underwent hepatic resection or radiofrequency ablation^52^ and among patients who underwent liver transplantation^53^; in contrast, no such associations were found in two other studies^22^,^28^, but this might be due to a limited number of HCC cases and lack of statistical power. Our findings support the idea that HCV-induced liver damage is caused by inflammatory infiltration initiated by immune responses. In this study, we found that the SNPs rs368234815, rs8099917, and rs12979860 were significantly associated with spontaneous clearance of HCV RNA. However, among subjects with detectable HCV RNA with HCV genotype 1 infection, the odds ratio for HCC risk was only1.7 (0.88–3.3) for rs368234815, despite the correlation between rs368234815 and rs8099917 being 0.76 and that between rs368234815 and rs12979860 0.92. Whether other functional variants play a role in HCC risk needs to be examined. We found that two SNPs, rs8099917 and rs12979860, that were associated with spontaneous HCV RNA clearance were also associated with risk of HCC. However, this association was only seen in patients with HCV genotype 1 infection, suggesting that the host-virus interaction might be important for liver disease progression.

It is well documented that SNPs near *IFNL3* play significant roles in the treatment response in chronic hepatitis C patients with genotype 1 infection^14^,^15^,^16^. However, reports on the association of different variants and treatment responses in patients with HCV genotype non-1 have been controversial^18^,^54^,^55^,^56^,^57^. Recently, more and more evidence showed the variants seem to have predictive value on treatment response of patients with genotype 2 or 3 as well as those infected by HCV genotype 1^54^,^55^,^56^,^57^. It will be interesting to examine which HCV genotypes are associated with spontaneous HCV RNA clearance. However, only participants with detectable HCV RNA can be differentiated into different HCV genotypes. The viral genotype data was not available for those who had undetectable HCV RNA.

The autoimmune hepatitis, toxic hepatitis, and primary biliary cirrhosis were not excluded in this study because the data was not available. However, these liver diseases are less important than hepatocellular carcinoma in Taiwan and there’s still no report to imply that the IFNL genetic variants were associated with these diseases. Thus, the findings in our study influenced by these liver diseases will be limited.

Direct acting antiviral agents (non-interferon-based) are becoming the standard means of treating HCV infection. Although the new antiviral drugs are impressive in terms of their high efficacy, short treatment course, oral administration, and good tolerance, they are extremely expensive. Not all patients with HCV infection who pay for treatment themselves and not all health insurance companies can afford the high price. It is therefore essential to stratify high risk patients who will have priority for new therapies. Our study found that SNPs near *IFNL3* and *IFNL4* were associated with spontaneous HCV RNA clearance. In addition, variants associated with less likelihood of spontaneous HCV RNA clearance (the host clears the virus itself) were associated with increased risk for HCC in subjects infected by HCV genotype 1. This suggests that it might be clinically relevant to test genetic polymorphism in patients with HCV infection, as patients who carry the unfavorable genotype may not clear HCV virus by themselves and are less likely to respond to interferon-based therapy. This is specially relevant in patients infected with HCV genotype 1, in whom we found an increased risk of developing HCC. This suggests that patients who carry an unfavorable genotype (rs12980275: AG/GG; rs12979860: CT/TT; rs4803222: CG/GG; rs8113007: TA/AA; and rs8099917: TG/GG) should be considered for the new direct acting antiviral agents, rather than for conventional interferon-based therapy^58^,^59^.

In summary, we found that host variants near *IFNL3* and *IFNL4* are associated with spontaneous HCV RNA clearance and that individuals who carry the minor allele of polymorphisms near the *IFNL3* gene are less likely to show spontaneous HCV clearance and have increased risk for HCC, particularly in those with HCV genotype 1 infection.

## Additional Information

**How to cite this article**: Lee, M.-H. *et al.* Polymorphisms near the *IFNL3* Gene Associated with HCV RNA Spontaneous Clearance and Hepatocellular Carcinoma Risk. *Sci. Rep.* **5**, 17030; doi: 10.1038/srep17030 (2015).

## Supplementary Material

Supplementary Information

## Figures and Tables

**Table 1:** Baseline characteristics of individuals with spontaneous HCV RNA clearance or HCV RNA persistence.

Baseline characteristic	Total population (N = 889)	Individuals with spontaneous HCV RNA clearance (N = 294), n (%)		Individuals with HCV RNA persistence (N = 595), n (%)		P value
Sex						
Male	396 (44.5%)	97	(33.0%)	299	(50.3%)	<0.001
Female	493 (55.5%)	197	(67.0%)	296	(49.7%)	
Age (years)						
30–39	152 (17.1%)	58	(19.7%)	94	(15.8%)	0.165
40–49	204 (22.9%)	75	(25.5%)	129	(21.7%)	
50–59	359 (40.4%)	107	(36.4%)	252	(42.4%)	
≥60	174 (19.6%)	54	(18.4%)	120	(20.2%)	
BMI (kg/m^2^)						
<23	340 (38.3%)	94	(32.0%)	246	(41.4%)	0.016
23–<25	195 (22.0%)	76	(26.9%)	119	(20.0%)	
25+	353 (39.8%)	124	(42.2%)	229	(38.6%)	
Triglycerides (mg/dl)						
<150	682 (77.5%)	187	(65.4%)	495	(83.3%)	<0.001
≥150	198 (22.5%)	99	(34.6%)	99	(16.7%)	
Unknown	9	8		1		
Cholesterol (mg/dl)						
<240	798 (90.8%)	240	(83.9%)	558	(94.1%)	<0.001
≥240	81 (9.2%)	46	(16.1%)	35	(5.9%)	
Unknown	10	8		2		
AST (U/L)						
<15	230 (25.9%)	134	(45.6%)	96	(16.1%)	<0.001
15–45	513 (57.7%)	140	(47.6%)	373	(62.7%)	
≥45	146 (16.4%)	20	(6.8%)	126	(21.2%)	
ALT (U/L)						
<15	359 (40.4%)	187	(63.6%)	172	(28.9%)	<0.001
15–45	381 (42.9%)	88	(29.9%)	293	(49.2%)	
≥45	149 (16.8%)	19	(6.5%)	130	(21.8%)	

BMI, body mass index; ALT, alanine aminotransferase; AST, aspartate aminotransferase.

**Table 2:** Baseline characteristics associated with spontaneous HCV RNA clearance.

Baseline characteristic	Crude odds ratio (95% CI)	P value	Adjusted odds ratio (95% CI)	P value
Sex				
Male	1.00		1.00	
Female	2.05 (1.53–2.75)	<0.001	1.99 (1.43–2.77)	<0.001
Age (years)				
30–39	1.00			
40–49	0.94 (0.61–1.45)	0.79		
50–59	0.69 (0.46–1.02)	0.07		
≥60	0.73 (0.46–1.15)	0.18		
BMI (kg/m^2^)				
<23	1.00		1.00	
23–<25	1.67 (1.15–2.43)	0.007	2.00(1.30–3.08)	0.002
25+	1.42 (1.03–1.96)	0.03	1.57 (1.08–2.28)	0.019
Triglycerides (mg/dl)				
<150	1.00		1.00	
≥150	2.65 (1.91–3.67)	<0.001	2.17 (1.50–3.15)	<0.001
Total cholesterol (mg/dl)				
<240	1.00		1.00	
≥240	3.06 (1.92–4.86)	<0.001	2.46 (1.47–4.13)	<0.001
AST (U/L)				
<15	1.00		1.00	
15–45	0.28 (0.20–0.39)	<0.001	0.42 (0.29–0.62)	<0.001
≥45	0.12 (0.07–0.20)	<0.001	0.34 (0.16–0.74)	0.006
ALT (U/L)				
<15	1.00		1.00	
15–45	0.29 (0.21–0.39)	<0.001	0.38 (0.26–0.55)	<0.001
≥45	0.14 (0.08–0.23)	<0.001	0.23 (0.11–0.48)	<0.001

BMI, body mass index; ALT, alanine aminotransferase; AST, aspartate aminotransferase.

**Table 3:** Eleven single nucleotide polymorphisms (SNP) significantly associated with spontaneous HCV RNA clearance.

SNP	Total population (N=889), n (%)	Individuals with spontaneous HCV RNA clearance (N = 294), n (%)	Individuals with HCV RNA persistence (N = 595), n (%)	P value*
rs35790907				
AA	793 (89.2)	274 (93.2)	519 (87.2)	0.0289*
AT/TT	96 (10.8)	20 (6.8)	76 (12.8)	
rs12980275				
AA	800 (90.0)	275 (93.5)	525 (88.2)	0.0382*
AG/GG	89 (10.0)	19 (6.5)	70 (11.8)	
rs4803217				
CC	803 (90.3)	277 (94.2)	526 (88.4)	0.0280*
CA/AA	86 (9.7)	17 (5.8)	69 (11.6)	
rs11881222				
AA	803 (90.3)	277 (94.2)	526 (88.4)	0.0280*
AG/GG	86 (9.7)	17 (5.8)	69 (11.6)	
rs12979860				
CC	798 (90.1)	276 (94.2)	522 (88.0)	0.0280*
CT/TT	88 (9.9)	17 (5.8)	71 (12.0)	
rs4803222				
CC	803 (90.3)	277 (94.2)	526 (88.4)	0.0280*
CG/GG	86 (9.7)	17 (5.8)	69 (11.6)	
rs8109889				
CC	803 (90.6)	277 (94.5)	526 (88.7)	0.0280*
CT/TT	83 (9.4)	16 (5.5)	67 (11.3)	
rs8113007				
TT	802 (90.2)	276 (93.9)	526 (88.4)	0.0324*
TA/AA	87 (9.8)	18 (6.1)	69 (11.6)	
rs8099917				
TT	815 (91.7)	279 (94.9)	536 (90.1)	0.0382*
TG/GG	74 (8.3)	15 (5.1)	59 (9.9)	
rs73050457				
CC	835 (93.9)	286 (97.3)	549 (92.3)	0.0280*
CT	54 (6.1)	8 (2.72)	46 (7.7)	
rs368234815				
TT/TT	637 (91.4)	268 (94.7)	369 (89.1)	0.0324*
TT/ΔGorΔG/ΔG	60 (8.6)	15 (5.3)	45 (10.9)	

^*^p value corrected by the false discovery rate (FDR).

**Table 4:** Associated odds ratios of the 11 single nucleotide polymorphisms (SNPs) and spontaneous HCV RNA clearance.

SNP	Crude odds ratio (95% CI)		P value	Adjusted odds ratio (95% CI)*		P value
rs35790907						
AT/TT	1.00			1.00		
AA	2.01	(1.2–3.35)	0.008	2.35	(1.37–4.04)	0.002
rs12980275						
AG/GG	1.00			1.00		
AA	1.93	(1.14–3.27)	0.015	2.23	(1.27–3.91)	0.005
rs4803217						
CA/AA	1.00			1.00		
CC	2.14	(1.23–3.71)	0.007	2.55	(1.42–4.6)	0.002
rs11881222						
AG/GG	1.00			1.00		
AA	2.14	(1.23–3.71)	0.007	2.54	(1.41–4.58)	0.002
rs12979860						
CT/TT	1.00			1.00		
CC	2.21	(1.28–3.82)	0.005	2.65	(1.47–4.76)	0.001
rs4803222						
CG/GG	1.00			1.00		
CC	2.14	(1.23–3.71)	0.007	2.54	(1.41–4.58)	0.002
rs8109889						
CT/TT	1.00			1.00		
CC	2.21	(1.25–3.88)	0.006	2.53	(1.38–4.63)	0.003
rs8113007						
TA/AA	1.00			1.00		
TT	2.01	(1.17–3.45)	0.011	2.36	(1.33–4.21)	0.003
rs8099917						
TG/GG	1.00			1.00		
TT	2.05	(1.14–3.68)	0.016	2.42	(1.29–4.53)	0.006
rs73050457						
CT/TT	1.00			1.00		
CC	3.00	(1.4–6.43)	0.005	3.12	(1.41–6.87)	0.005
rs368234815						
TT/ΔGorΔG/ΔG	1.00			1.00		
TT/TT	2.18	(1.19–3.99)	0.012	2.85	(1.48–5.46)	0.002

^*^adjusted for sex, body mass index, serum levels of triglycerides and total cholesterol, and serum levels of AST and ALT.

**Table 5:** Associated risks for hepatocellular carcinoma of single nucleotide polymorphisms (SNPs) near the *IFNL2*, *IFNL3*, and *IFNL4* genes stratified by HCV genotype.

SNP	Total participants (n = 999)	Participants with HCV genotype 1 (n = 541)	P value	Participants with HCV genotype non-1 (394)	P value
	Numbers of cases/controls	Odds ratio (95% CI)*		Odds ratio (95% CI)*	
rs35790907					
AA	413/452	1.00		1.00	
AT/TT	68/64	1.59 (0.92–2.75)	0.0983	0.9 (0.42–1.95)	0.7968
rs12980275					
AA	415/455	1.00		1.00	
AG/GG	67/61	1.81 (1.03–3.16)	0.0379	0.67 (0.31–1.44)	0.3063
rs4803217					
CC	414/456	1.00		1.00	
CA/AA	65/60	1.72 (0.98–3.02)	0.0573	0.61 (0.28–1.33)	0.2154
rs11881222					
AA	414/456	1.00		1.00	
AG/GG	64/60	1.70 (0.96–2.99)	0.0670	0.65 (0.30–1.40)	0.2741
rs12979860					
CC	411/452	1.00		1.00	
CT/TT	69/62	1.71 (0.99–2.97)	0.0543	0.67 (0.32–1.44)	0.3046
rs4803222					
CC	414/456	1.00		1.00	
CG/GG	65/60	1.78 (1.01–3.11)	0.045	0.61 (0.28–1.33)	0.2154
rs8109889					
CC	419/457	1.00		1.00	
CT/TT	62/59	1.51 (0.86–2.66)	0.1505	0.71 (0.33–1.56)	0.3995
rs8113007					
TT	415/456	1.00		1.00	
TA/AA	66/60	1.76 (1.01–3.09)	0.0476	0.67 (0.31–1.44)	0.3040
rs8099917					
TT	419/464	1.00		1.00	
TG/GG	62/52	1.83 (1.01–3.29)	0.0453	0.89 (0.40–1.98)	0.7812
rs73050457					
CC	436/475	1.00		1.00	
CT/TT	45/41	1.77 (0.9–3.5)	0.0989	0.96 (0.39–2.39)	0.9278
rs368234815					
TT/TT	348/345	1.00		1.00	
TT/ΔGorΔG/ΔG	53/41	1.68 (0.86–3.25)	0.1269	1.19 (0.45–3.17)	0.7296

^*^adjusted for age, sex, serum HCV RNA levels, and serum levels of ALT.
