# Supplementary material for: Polymorphisms near the IFNL3 Gene Associated with HCV RNA Spontaneous Clearance and Hepatocellular Carcinoma Risk
Source: Sci Rep. 2015 Nov 25;5:17030. doi: 10.1038/srep17030 (PMC4658500; doi:10.1038/srep17030)
Supplement: Supplementary Information [file srep17030-s1.doc]

Supplementary

manuscript title: **Polymorphisms Near the *IFNL3* Gene Associated with HCV RNA Spontaneous Clearance and Hepatocellular Carcinoma Risk**

Mei-Hsuan Lee, Hwai-I Yang, Sheng-Nan Lu, Yu-Ju Lin, Chin-Lan Jen, Kang-Hsuan Wong, Soa-Yu Chan, Liang-Chun Chen, Li-Yu Wang, Gilbert L’Italien, Yong Yuan, and Chien-Jen Chen

**Supplementary Table 1**

**The 29 single nucleotide polymorphisms examined for associations with spontaneous HCV RNA clearance**

| SNP | Total population  (N=889), n (%) | Individuals with spontaneous HCV RNA clearance (N=294), n (%) | Individuals with HCV RNA persistence (N=595), n (%) | P value |
| --- | --- | --- | --- | --- |
| rs11083515 |  |  |  |  |
| AA | 249 (28.0) | 88 (29.9) | 161 (27.1) | 0.7579 |
| AG | 441 (49.6) | 143 (48.6) | 298 (50.1) |  |
| GG | 199 (22.4) | 63 (21.4) | 136 (22.9) |  |
| rs11879005 |  |  |  |  |
| CC | 320 (36.0) | 112 (38.1) | 208 (35.0) | 0. 7579 |
| CT | 421 (47.4) | 136 (46.3) | 285 (47.9) |  |
| TT | 148 (16.6) | 46 (15.7) | 102 (17.1) |  |
| rs12975799 |  |  |  |  |
| GG | 320 (36.0) | 113 (38.4) | 207 (34.8) | 0.7083 |
| GA | 420 (47.3) | 135 (45.9) | 285 (48.0) |  |
| AA | 148 (16.7) | 46 (15.7) | 102 (17.2) |  |
| rs11883239 |  |  |  |  |
| GG | 309 (34.8) | 108 (36.7) | 201 (33.8) | 0.7083 |
| GA | 426 (47.9) | 140 (47.6) | 286 (48.1) |  |
| AA | 154 (17.3) | 46 (15.7) | 108 (18.2) |  |
| rs955155 |  |  |  |  |
| CC | 813 (91.6) | 278 (94.6) | 535 (90.1) | 0.0569 |
| CT/TT | 75 (8.4) | 16 (5.4) | 59 (9.9) |  |
| rs35790907 |  |  |  |  |
| AA | 793 (89.2) | 274 (93.2) | 519 (87.2) | 0.0289* |
| AT/TT | 96 (10.8) | 20 (6.8) | 76 (12.8) |  |
| rs12980275 |  |  |  |  |
| AA | 800 (90.0) | 275 (93.5) | 525 (88.2) | 0.0382* |
| AG/GG | 89 (10.0) | 19 (6.5) | 70 (11.8) |  |
| rs12982533 |  |  |  |  |
| TT | 788 (88.6) | 270 (91.8) | 518 (87.1) | 0.0774 |
| TC/CC | 92 (11.4) | 24 (8.2) | 77 (12.9) |  |
| rs4803217 |  |  |  |  |
| CC | 803 (90.3) | 277 (94.2) | 526 (88.4) | 0.0280* |
| CA/AA | 86 (9.7) | 17 (5.8) | 69 (11.6) |  |
| rs11881222 |  |  |  |  |
| AA | 803 (90.3) | 277 (94.2) | 526 (88.4) | 0.0280* |
| AG/GG | 86 (9.7) | 17 (5.8) | 69 (11.6) |  |
| rs12979860 |  |  |  |  |
| CC | 798 (90.1) | 276 (94.2) | 522 (88.0) | 0.0280* |
| CT/TT | 88 (9.9) | 17 (5.8) | 71 (12.0) |  |
| rs4803222 |  |  |  |  |
| CC | 803 (90.3) | 277 (94.2) | 526 (88.4) | 0.0280* |
| CG/GG | 86 (9.7) | 17 (5.8) | 69 (11.6) |  |
| rs1549928 |  |  |  |  |
| AA | 729 (82.0) | 237 (80.6) | 492 (82.7) | 0.6265 |
| AG/GG | 160 (18.0) | 57 (19.4) | 103 (17.3) |  |
| rs8109889 |  |  |  |  |
| CC | 803 (90.6) | 277 (94.5) | 526 (88.7) | 0.0280* |
| CT/TT | 83 (9.4) | 16 (5.5) | 67 (11.3) |  |
| rs8113007 |  |  |  |  |
| TT | 802 (90.2) | 276 (93.9) | 526 (88.4) | 0.0324* |
| TA/AA | 87 (9.8) | 18 (6.1) | 69 (11.6) |  |
| rs8099917 |  |  |  |  |
| TT | 815 (91.7) | 279 (94.9) | 536 (90.1) | 0.0382* |
| TG/GG | 74 (8.3) | 15 (5.1) | 59 (9.9) |  |
| rs73050457 |  |  |  |  |
| CC | 835 (93.9) | 286 (97.3) | 549 (92.3) | 0.0280* |
| CT | 54 (6.1) | 8 (2.72) | 46 (7.7) |  |
| rs10853728 |  |  |  |  |
| GG | 588 (66.3) | 204 (69.9) | 384 (64.5) | 0.3218 |
| GC | 264 (29.8) | 75 (25.7) | 189 (31.8) |  |
| CC | 35 (3.9) | 13 (4.5) | 22 (3.7) |  |
| rs35963157 |  |  |  |  |
| AA | 464 (52.2) | 149 (50.7) | 315 (52.9) | 0.8217 |
| AC | 367 (41.3) | 124 (42.2) | 243 (40.8) |  |
| CC | 58 (6.5) | 21 (7.1) | 37 (6.2) |  |
| rs12980602 |  |  |  |  |
| TT | 765 (86.1) | 255 (86.7) | 510 (85.7) | 0.7579 |
| TC/CC | 124 (13.9) | 39 (13.3) | 85 (14.3) |  |
| rs62120535 |  |  |  |  |
| GG | 832 (93.6) | 270 (91.8) | 562 (94.5) | 0.2775 |
| GC/CC | 57 (6.4) | 24 (8.2) | 33 (5.5) |  |
| rs62120542 |  |  |  |  |
| GG | 773 (87.2) | 253 (86.1) | 520 (87.8) | 0.6265 |
| GC/CC | 113 (12.8) | 41 (13.9) | 72 (12.2) |  |
| rs7359950 |  |  |  |  |
| CC | 778 (87.5) | 253 (86.1) | 525 (88.2) | 0.5414 |
| CT/TT | 111 (12.5) | 41 (14.0) | 70 (11.8) |  |
| rs2082419 |  |  |  |  |
| TT | 777 (87.5) | 252 (86.0) | 525 (88.2) | 0.5414 |
| TA/AA | 111 (12.5) | 41 (14.0) | 70 (11.8) |  |
| rs56655605 |  |  |  |  |
| CC | 833 (93.7) | 271 (92.2) | 562 (94.5) | 0.3218 |
| CT/TT | 56 (6.3) | 23 (7.8) | 33 (5.5) |  |
| rs62122862 |  |  |  |  |
| TT | 834 (93.8) | 276 (93.9) | 558 (93.8) | 0.9554 |
| TC/CC | 55 (6.2) | 18 (6.1) | 37 (6.2) |  |
| rs11665818 |  |  |  |  |
| GG | 833 (93.7) | 271 (92.2) | 562 (94.5) | 0.3218 |
| GA/AA | 56 (6.3) | 23 (7.8) | 33 (5.5) |  |
| rs60885838 |  |  |  |  |
| AA | 835 (94.0) | 277 (94.2) | 558 (93.9) | 0.9002 |
| AG/GG | 53 (6.0) | 17 (5.8) | 36 (6.1) |  |
| rs368234815 |  |  |  |  |
| TT/TT | 637 (91.4) | 268 (94.7) | 369 (89.1) | 0.0324* |
| TT/ΔGorΔG/ΔG | 60 (8.6) | 15 (5.3) | 45 (10.9) |  |

^*^p value corrected by the false discovery rate (FDR)
